# Supplementary material for: Data-efficient biomedical in-context learning: a diversity-enhanced submodular perspective
Source: NPJ Artif Intell. 2026 Jun 11;2(1):69. doi: 10.1038/s44387-026-00123-0 (PMC13612249; doi:10.1038/s44387-026-00123-0)
Supplement: Supplementary file 1 [file 44387_2026_123_MOESM1_ESM.pdf]

1. Implementation Details

We set k_1 to 100, consistent with established studies, and use $k = 3$ as the default value. The impact of k is further analyzed in our sensitivity analysis.

To reduce formatting errors in the inference results generated by LLMs, we found that performing a warm-up procedure using Low-Rank Adaptation (LoRA) [1] between pretraining and ICL inference is beneficial. For LoRA, we set the rank to 64 and alpha to 32. We fine-tuned the models for 4,000 steps on ChemProt, 2,000 steps on DDI, and 2,000 steps on HealthAdvice. All fine-tuning used the AdamW optimizer with a learning rate of 1e-5. The batch size was 2 during training and 4 during inference across all datasets.

All experiments were conducted on a single NVIDIA A100 GPU with 40GB of memory. Results are reported as the average over five runs.

2. Prompt Templates

We present the prompt templates utilized for different NLP tasks below, where the task description is listed in Supplementary Table 1.

```
### Instruction:
<Task Description>

### Examples:
#Input: <Demonstraion#1>
#Response: <Label#1>
...
#Input: <Demonstraion#k>
#Response: <Label#k>

### Input:
<Test Query>

### Response:
<Inference Result>
```

Supplementary Table 1. Detailed instructions for each task.

Task	Description
NER	Please do a named entity recognition task. You need to accurately recognize chemical or genetic words or terms given the input sentence. The response should only be entities following the form shown in the original sentences. If the given sentence does not include these kinds of entities, just output None.
RE	Please do a relation extraction task. You need to extract the relationship between the given head entity and the tail entity. The response should be in a predefined set: {'mechanism', 'effect, 'advice', 'int', 'none'}.<br>mechanism: this type is used to annotate drug-drug interactions that are described by their pharmacokinetic mechanism.<br>effect: this type is used to annotate drug-drug interactions describing an effect or a pharmacodynamic mechanism.<br>advice: this type is used when a recommendation or advice regarding a drug interaction is given.<br>int: this type is used when a drug-durg interaction appears in the text without providing any additional information.<br>none: there are no drug-drug interactions.
TC	Please do a classification task. You need to classify what type advice the input sentence is. The response should be in pre-defined set: ('no', 'weak', 'strong').

3. Algorithm

The algorithm details of our method, including the retrieval and ranking stages, are presented in Supplementary Algorithm 1.

Supplementary Algorithm 1 The Dual-Div Algorithm

Input: Corpus set V, monotone submodular function f, the cardinality limit k_1 and k
Output: A subset $T \subseteq S \subseteq V$ with $|T| \leq k$ and $|S| \leq k_1$

Phase 1 – Diversity-enhanced Retrieval

Initialize $S \leftarrow \emptyset$ and an empty max-heap $\mathcal{H}$ ▷ $\mathcal{H}$ stores pairs (x, δ) sorted by δ
for each element $x \in V$ **do**
    $\delta_x \leftarrow f(\{x\}) - f(\emptyset)$ ▷ Initial marginal gain
    $\mathcal{H}.\text{PUSH}((x, \delta_x))$
end for
for $i = 1$ to k_1 **do**
    if $\mathcal{H}$ is empty **then**
        break ▷ No elements left to add
    end if
    $(x_{\text{top}}, \delta_{\text{top}}) \leftarrow \mathcal{H}.\text{POP}()$ ▷ Element with max δ
    Compute current marginal gain: $\delta_{\text{new}} \leftarrow f(S \cup \{x_{\text{top}}\}) - f(S)$
    if $\mathcal{H}$ is not empty **then**
        $(x_{\text{next}}, \delta_{\text{next}}) \leftarrow \mathcal{H}.\text{PEEK}()$ ▷ Next best element in heap
        if $\delta_{\text{new}} \geq \delta_{\text{next}}$ **then**
            $S \leftarrow S \cup \{x_{\text{top}}\}$ ▷ Add x_{top} to solution
        else
            $\mathcal{H}.\text{PUSH}((x_{\text{top}}, \delta_{\text{new}}))$ ▷ Reinsert with updated gain
        end if
    else
        $S \leftarrow S \cup \{x_{\text{top}}\}$ ▷ Add last remaining element
    end if
end for
return S

Phase 2 – Diversity-enhanced Ranking

Initialize $T \leftarrow \emptyset$
for $x \in S^*$ **do**
    Compute $f(\{x_i\} \mid Q) = f(\{x_i\} \cup Q) - f(Q)$ ▷ Initial marginal gain
end for
for $i = 1$ to k **do**
    Select $x_{\text{top}} = \arg\max_{x \in S^* \setminus T} f(\{x_i\} \mid Q)$
    Update $T \leftarrow T \cup \{x_{\text{top}}\}$ ▷ Add x_{top} to solution
end for
return T

4. More Experimental Results

4.1. ICL Performance

We supplement the in-context learning results of all methods for different tasks with BMRetriever and MedCPT as the retriever models in Supplementary Table 2 and Supplementary Table 3 separately.

4.2. Effect of Example Ordering

For the sensitivity analysis regarding the permutation ordering within LLM's input prompts, we supplement the violin plots of all possible permutations of the examples in the final set T, using BMRetriever and MedCPT as the retriever, in Supplementary Figure 1 and Supplementary Figure 2 separately.

Supplementary Table 2. ICL Performance on different NLP datasets using Llama 3.1 (8B) and Qwen 2.5 (7B) as inference LLMs and BMRetriever as the retriever (Diff rows report Dual-Div − Div-S3 as mean difference with 95% CI and paired t-test p-value from 10-fold cross-validation).

LLM	Method	ChemProt (NER)		DDI (RE)		HealthAdvice (TC)	
		acc.	macro-F1	acc.	macro-F1	acc.	macro-F1
Qwen 2.5	Random-Simliar	0.7118	0.6673	0.7499	0.2513	0.7592	0.2921
	Div-S3	0.7028	0.6881	0.7065	0.2689	0.7644	0.3350
	Div*-S3	**0.7364**	0.7220	**0.7756**	0.3049	**0.7667**	**0.3456**
	Div-S3*	0.7085	0.7051	0.7065	0.2689	0.7656	0.3390
	Dual-Div	0.7324	**0.7261**	0.7716	**0.3180**	**0.7667**	**0.3456**
	Diff of acc.	$[0.0260, 0.0410]^{***}$		$[0.0549, 0.0752]^{***}$		$[0.0012, 0.0064]^{*}$	
	Diff of macro-F1	$[0.0330, 0.0429]^{***}$		$[0.0243, 0.0487]^{***}$		$[-0.0024, 0.0219]$	
Llama 3.1	Random-Simliar	0.6815	0.6261	0.6678	0.2494	0.6751	0.2786
	Div-S3	0.6926	0.6343	0.6797	0.2582	0.6897	0.2830
	Div*-S3	0.7082	0.6493	**0.6950**	0.2844	**0.7615**	0.2874
	Div-S3*	0.7010	0.6521	0.6813	0.2862	0.7177	0.2883
	Dual-Div	**0.7095**	**0.6574**	0.6936	**0.2932**	0.7563	**0.2890**
	Diff of acc.	$[-0.0045, 0.0122]^{*}$		$[0.0023, 0.0217]^{*}$		$[0.0578, 0.0846]^{***}$	
	Diff of macro-F1	$[0.0086, 0.0269]^{**}$		$[0.0209, 0.0508]^{***}$		$[-0.0007, 0.0041]$	

Note: $^{*}p < 0.05$, $^{**}p < 0.01$, $^{***}p < 0.001$.

Supplementary Table 3. ICL Performance on different NLP datasets using Llama 3.1 (8B) and Qwen 2.5 (7B) as inference LLMs and MedCPT as the retriever (Diff rows report Dual-Div − Div-S3 as mean difference with 95% CI and paired t-test p-value from 10-fold cross-validation).

LLM	Method	ChemProt (NER)		DDI (RE)		HealthAdvice (TC)	
		acc.	macro-F1	acc.	macro-F1	acc.	macro-F1
Qwen 2.5	Random-Simliar	0.6864	0.6704	0.7559	0.2488	0.7446	0.2796
	Div-S3	**0.7421**	0.7064	0.7559	0.2789	0.7615	0.2923
	Div*-S3	**0.7421**	0.7064	**0.8070**	0.2588	0.7615	0.2963
	Div-S3*	0.7190	0.7119	0.7771	0.3005	**0.7620**	0.3039
	Dual-Div	0.7284	**0.7155**	0.7870	**0.3115**	0.7598	**0.3179**
	Diff of acc.	$[-0.0170, -0.0103]^{***}$		$[0.0220, 0.0400]^{*}$		$[-0.0069, 0.0035]$	
	Diff of macro-F1	$[0.0031, 0.0111]^{**}$		$[0.0303, 0.0526]^{***}$		$[-0.0121, 0.0282]$	
Llama 3.1	Random-Simliar	0.6351	0.5184	0.6966	0.2676	0.6230	0.2805
	Div-S3	0.6681	0.6225	**0.7440**	0.2839	0.7158	0.2917
	Div*-S3	**0.6695**	0.6228	0.7089	0.2983	0.7085	0.2954
	Div-S3*	0.6444	0.6510	0.7431	0.2898	**0.7558**	0.2889
	Dual-Div	0.6507	**0.6581**	0.7341	**0.3039**	0.7298	**0.2988**
	Diff of acc.	$[-0.0273, -0.0122]^{***}$		$[-0.0181, 0.0466]^{*}$		$[0.0169, 0.0203]^{***}$	
	Diff of macro-F1	$[0.0179, 0.0394]^{***}$		$[-0.0046, 0.0388]$		$[-0.0015, 0.0054]$	

Note: $^{*}p < 0.05$, $^{**}p < 0.01$, $^{***}p < 0.001$.

4.3. Case Study

Finally, we present case studies: the top demonstrations selected by Dual-Div and Div-S3 on the DDI and HealthAdvice datasets, shown in Supplementary Figure 3 and Supplementary Figure 4, respectively.

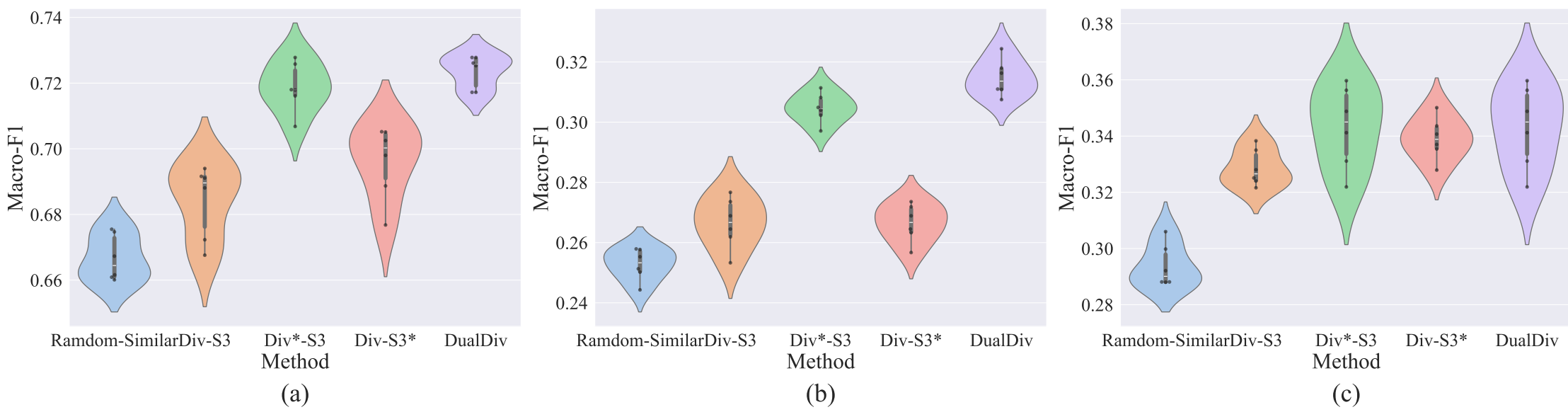

Supplementary Fig. 1: Sensitivity analysis regarding the permutation ordering in the LLM's prompts of the final demonstration set. Given BMRetriever as the retriever model, for each set of 3 demonstrations from different methods, we plot the violin graphics of macro-F1 scores of all 6 possible permutations on the (a) ChemProt, (b) DDI, and (c) HealthAdvice datasets.

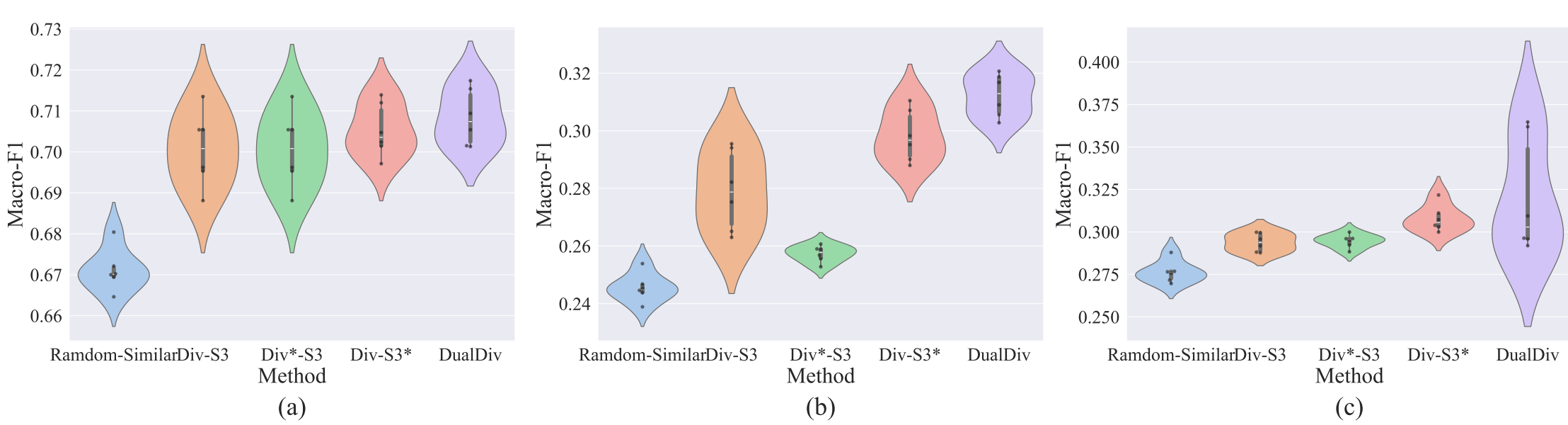

Supplementary Fig. 2: Sensitivity analysis regarding the permutation ordering in the LLM's prompts of the final demonstration set. Given MedCPT as the retriever model, for each set of 3 demonstrations from different methods, we plot the violin graphics of macro-F1 scores of all 6 possible permutations on the (a) ChemProt, (b) DDI, and (c) HealthAdvice datasets.

Ranked Demonstrations from *Dual-Div*

#Example 1: When used in therapeutic doses, @DRUG1$ had a modest effect on the pharmacokinetics of atorvastatin, carbamazepine, cetirizine, didanosine, efavirenz, fluconazole, indinavir, midazolam, rifabutin, sildenafil, theophylline (intravenous and oral), triazolam, trimethoprim/sulfamethoxazole or @DRUG2$. What is the relationship between @DRUG1$ and @DRUG2$?
#Response: mechanism

#Example 2: Concomitant medications were grouped as @DRUG1$, oral anticoagulants, calcium channel blockers, beta blockers, cardiac glycosides, inducers of CYP3A4, substrates and inhibitors of CYP3A4, substrates and inhibitors of P-glycoprotein, nitrates, @DRUG2$, loop diuretics, potassium sparing diuretics, thiazide diuretics, substrates and inhibitors of tubular organic cation transport, and QTc-prolonging drugs. What is the relationship between @DRUG1$ and @DRUG2$?
#Response: none

#Example 3: Studies have shown that @DRUG1$ does not have clinically significant interactions with other drugs metabolized by the cytochrome P450 system, such as warfarin, antipyrine, indomethacin, ibuprofen, phenytoin, propranolol, prednisone, diazepam, clarithromycin, or @DRUG2$ in healthy subjects. What is the relationship between @DRUG1$ and @DRUG2$?
#Response: none

Ranked Demonstrations from *Div-S3*

#Example 1: When used in therapeutic doses, @DRUG1$ had a modest effect on the pharmacokinetics of atorvastatin, carbamazepine, cetirizine, didanosine, efavirenz, @DRUG2$, indinavir, midazolam, rifabutin, sildenafil, theophylline (intravenous and oral), triazolam, trimethoprim/sulfamethoxazole or zidovudine. What is the relationship between @DRUG1$ and @DRUG2$?
#Response: mechanism

#Example 2: When used in therapeutic doses, @DRUG1$ had a modest effect on the pharmacokinetics of atorvastatin, carbamazepine, cetirizine, didanosine, efavirenz, fluconazole, indinavir, midazolam, @DRUG2$, sildenafil, theophylline (intravenous and oral), triazolam, trimethoprim/sulfamethoxazole or zidovudine. What is the relationship between @DRUG1$ and @DRUG2$?
#Response: mechanism

#Example 3: When used in therapeutic doses, @DRUG1$ had a modest effect on the pharmacokinetics of atorvastatin, carbamazepine, cetirizine, didanosine, efavirenz, fluconazole, indinavir, midazolam, rifabutin, sildenafil, theophylline (intravenous and oral), triazolam, trimethoprim/sulfamethoxazole or @DRUG2$. What is the relationship between @DRUG1$ and @DRUG2$?
#Response: mechanism

Supplementary Fig. 3: Case study of ranked demonstrations on the DDI dataset for RE task from (diversity-enhanced) *Dual-Div* and *Div-S3*, with BGE-Large as the retriever and Qwen 2.5 as the inference LLM.

Ranked Demonstrations from *Dual-Div*

#Example 1: Although not without risk, it is generally a simple procedure leading to satisfactory weight loss, improvement in co-morbidities, and consequent reduction of the perioperative mortality and morbidity rates associated with surgery.
#Response: weak

#Example 2: This study provides the strongest evidence to date that sustained, carefully planned, differentiated literacy instruction supported by technology and using the best assessments and research-based strategies available is more likely to be effective than what is typically seen in classrooms.
#Response: no

#Example 3: There are several possible reasons for the apparent difference between children and adults: it may reflect population differences (the child carriers of the GSV were from France and the UK, the adults primarily from Nordic countries); it may reflect cohort ascertainment, for instance that the child cohorts did not include overweight or mildly obese subjects – it is notable, however, that the deletion was not reported at a comparable frequency in cohorts of children with common obesity [16]; it may reflect a genuine attenuation of the effect of the deletion in adults, so that impact of the GSV on obesity becomes less pronounced with increasing age; or the severe obesity observed in children may have been triggered by an aspect of the modern obesogenic environment that was experienced to a lesser degree by older subjects.
#Response: no

Ranked Demonstrations from *Div-S3*

#Example 1: This study provides the strongest evidence to date that sustained, carefully planned, differentiated literacy instruction supported by technology and using the best assessments and research-based strategies available is more likely to be effective than what is typically seen in classrooms.
#Response: no

#Example 2: There are several possible reasons for the apparent difference between children and adults: it may reflect population differences (the child carriers of the GSV were from France and the UK, the adults primarily from Nordic countries); it may reflect cohort ascertainment, for instance that the child cohorts did not include overweight or mildly obese subjects – it is notable, however, that the deletion was not reported at a comparable frequency in cohorts of children with common obesity [16]; it may reflect a genuine attenuation of the effect of the deletion in adults, so that impact of the GSV on obesity becomes less pronounced with increasing age; or the severe obesity observed in children may have been triggered by an aspect of the modern obesogenic environment that was experienced to a lesser degree by older subjects.
#Response: no

#Example 3:These observations are also in line with the previous findings [6,7,9,10,11,12].
#Response: no

Supplementary Fig. 4: Case study of ranked demonstrations on the HealthAdvice dataset for TC task from (diversity-enhanced) *Dual-Div* and *Div-S3*, with BGE-Large as the retriever and Qwen 2.5 as the inference LLM.

References

1. Edward J Hu, yelong shen, Phillip Wallis, Zeyuan Allen-Zhu, Yuanzhi Li, Shean Wang, Lu Wang, and Weizhu Chen. LoRA: Low-rank adaptation of large language models. In *International Conference on Learning Representations*, 2022.
